# Mitochondrial Quality Control: A Pathophysiological Mechanism and Therapeutic Target for Stroke

**DOI:** 10.3389/fnmol.2021.786099

**Published:** 2022-01-28

**Authors:** Miaoxian Yang, Yu He, Shuixiang Deng, Lei Xiao, Mi Tian, Yuewen Xin, Chaocheng Lu, Feng Zhao, Ye Gong

**Affiliations:** ^1^Department of Critical Care Medicine, Huashan Hospital, Fudan University, Shanghai, China; ^2^The State Key Laboratory of Medical Neurobiology, MOE Frontiers Center for Brain Science, The Institutes of Brain Science, Fudan University, Shanghai, China; ^3^Department of Neurosurgery, Huashan Hospital, Fudan University, Shanghai, China

**Keywords:** stroke, mitochondrial quality control, fission, fusion, mitophagy, neurovascular unit

## Abstract

Stroke is a devastating disease with high mortality and disability rates. Previous research has established that mitochondria, as major regulators, are both influenced by stroke, and further regulated the development of poststroke injury. Mitochondria are involved in several biological processes such as energy generation, calcium homeostasis, immune response, apoptosis regulation, and reactive oxygen species (ROS) generation. Meanwhile, mitochondria can evolve into various quality control systems, including mitochondrial dynamics (fission and fusion) and mitophagy, to maintain the homeostasis of the mitochondrial network. Various activities of mitochondrial fission and fusion are associated with mitochondrial integrity and neurological injury after stroke. Additionally, proper mitophagy seems to be neuroprotective for its effect on eliminating the damaged mitochondria, while excessive mitophagy disturbs energy generation and mitochondria-associated signal pathways. The balance between mitochondrial dynamics and mitophagy is more crucial than the absolute level of each process. A neurovascular unit (NVU) is a multidimensional system by which cells release multiple mediators and regulate diverse signaling pathways across the whole neurovascular network in a way with a high dynamic interaction. The turbulence of mitochondrial quality control (MQC) could lead to NVU dysfunctions, including neuron death, neuroglial activation, blood–brain barrier (BBB) disruption, and neuroinflammation. However, the exact changes and effects of MQC on the NVU after stroke have yet to be fully illustrated. In this review, we will discuss the updated mechanisms of MQC and the pathophysiology of mitochondrial dynamics and mitophagy after stroke. We highlight the regulation of MQC as a potential therapeutic target for both ischemic and hemorrhagic stroke.

## Introduction

Stroke is a devastating disease with high disability and mortality rates worldwide (GBD 2017 Causes of Death Collaborators, 2018). It is an acute cerebrovascular disease that is classified into ischemic stroke, hemorrhagic stroke, and transient ischemic attack. Stroke induces not only primary injury but also secondary brain injury that is triggered by mass effect, deprivation of glucose and oxygen, coagulation dysfunction, oxidative stress, excitotoxicity, inflammation, etc. (Shi et al., 2019). Unfortunately, a few effective medical or surgical therapies have been demonstrated to improve the prognosis of patients with stroke. Investigations into the pathophysiology and clinical treatment of patients with stroke remain necessary to improve their neurological outcomes.

Mitochondria are the double-membrane organelles that exist in most eukaryotic organisms. In addition to their critical role in energy conversion, mitochondria are involved in several biological processes, including calcium homeostasis, phospholipid biogenesis, innate immune, apoptosis regulation, and reactive oxygen species (ROS) generation (Bock and Tait, 2020; Zong et al., 2020). Interestingly, mitochondria evolve into various quality control systems, including mitochondrial dynamics, mitophagy, antioxidant defense, mitochondrial biogenesis, etc. (Tang et al., 2021). Mitochondrial dynamics, including fission and fusion, describe the ever-changing activities of mitochondrial shape, size, quantity, connectivity, and trafficking. Mitophagy is a process of mitochondrial autophagy that belongs to the form of macroautophagy. These quality control systems are multitiered, acting on the molecule, organelle, and cell levels to ensure the normal structure and function of the mitochondrial network and cellular homeostasis (Pickles et al., 2018).

Neurovascular unit (NVU) is a concept first formalized at the Stroke Progress Review Group meeting in 2001 (Sun et al., 2021). It emphasizes a holistic view among its main components, namely neurons, astroglia, microglia, and vessels (Sun et al., 2021). Physiologically, NVU plays a crucial role in regulating brain blood flow, blood–brain barrier (BBB), and neuroimmune responses (Liebner et al., 2018). Pathologically, the components of NVU were proven to perform different resistance, metabolic reactions, and immune responses to stroke, evidenced by intricate changes in (a) structural proteins, (b) neurotransmitters and enzymes, (c) inflammatory cytokines, and (d) neurotrophic and growth factors (Steliga et al., 2020).

**FIGURE 1:**
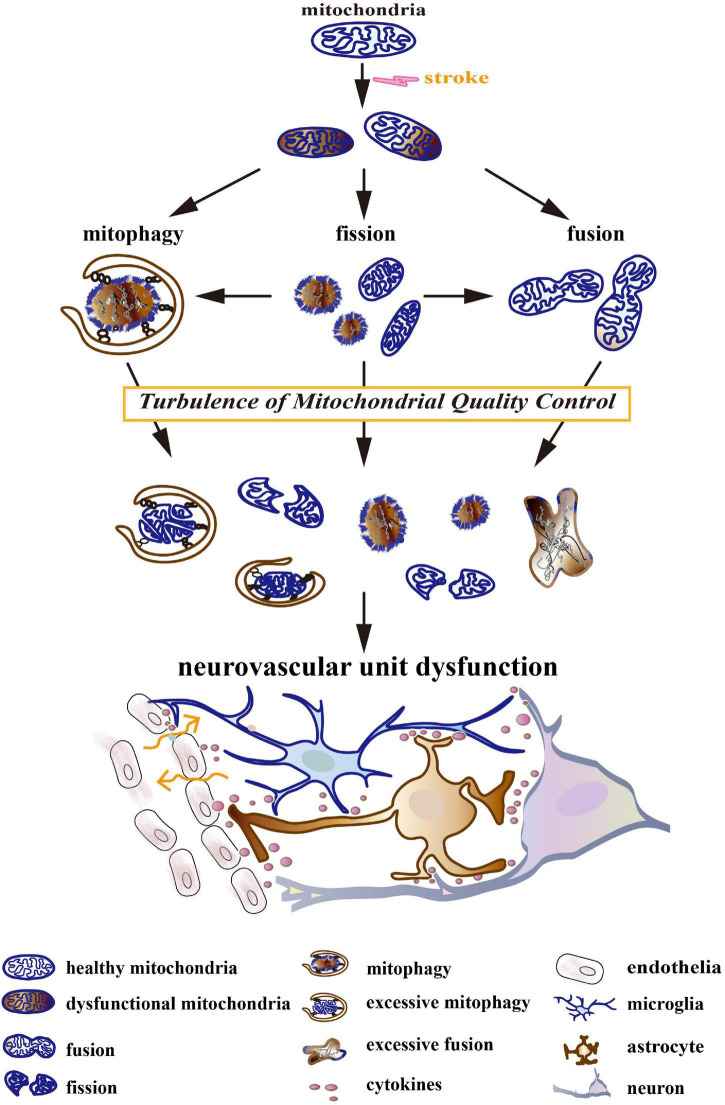
The turbulence of mitochondrial quality control (MQC) leads to neurovascular unit (NVU) dysfunction. Stroke induces a compromised availability of glucose and oxygen, mass effects, coagulation dysfunction, oxidative stress, excitotoxicity, inflammation, etc. They result in mitochondrial permeability transition, membrane depolarization, morphological disorder, Ca^2+^ overload, oxidative stress, and the release of mitochondrial cytochrome and DNA. Initially, MQC systems maintain the normal structure and function of the mitochondrial network by keeping the balance between mitochondrial dynamics (fission and fusion) and mitophagy. As the disease develops, MQC systems become turbulent. Increased fission, deficient fusion, and impaired/excessive mitophagy cause mitochondrial fragmentation, the accumulation of dysfunctional mitochondria, and the degradation of healthy mitochondria. The turbulence of the mitochondrial network further aggravates after-stroke neurovascular unit injury by triggering neuron death, neuroglial activation, blood–brain barrier (BBB) disruption, and neuroinflammation.

Turbulences of mitochondrial quality control (MQC) after stroke could lead to NVU dysfunctions, including neuron death, endothelial damage, neuroglial reaction, BBB disruption, and neuroinflammation (Figure 1; Cai et al., 2017; Salmina et al., 2021). The turbulences include increased fission, deficient fusion, and impaired or excessive mitophagy. Mechanically, MQC can be disturbed by a compromised availability of glucose and oxygen, hematoma mass effect, inflammation, and the release of cytotoxins after stroke (Keep et al., 2012; Qu et al., 2016). It leads to mitochondrial permeability transition, membrane depolarization, mitochondrial morphological disorder, Ca^2+^ overload, oxidative stress, and the release of mitochondrial cytochrome and DNA, which further aggravates NVU injury after stroke. Deficient fusion after stroke was associated with increased mitochondrial degradation and impaired ATP production (Roy et al., 2015; Adebayo et al., 2021). In turn, energetic status provides feedback to modulate the functions of MQC-associated proteins. MQC-driven proteins, particularly dynamin-related protein 1 (Drp1), mitofusin 2 (Mfn2), optic atrophy type 1 (OPA1), and PTEN-induced putative kinase protein 1 (PINK1), are dynamin-related GTPases whose activity mainly depends on GTP concentration (Takeda et al., 2018). Additionally, increased mitochondrial destruction after stroke could result in the overproduction of ROS and the impairment of antioxidant defense in a mitochondria-dependent pathway (Fischer et al., 2012; Held and Houtkooper, 2015). Oxidative stress was reported to trigger pro-inflammatory polarization of neuroglia, the apoptosis of neurons, and the injury of endothelia (Posada-Duque et al., 2014). Previous studies have observed that aberrant MQC in neurodegenerative diseases could cause a deficiency in Ca^2+^ homeostasis, which impaired synaptic function and plasticity (Mattson, 2007). Interestingly, in a previous study, different calcium concentrations variously remodeled mitochondrial morphology in astrocytes (Tan et al., 2011).

Given the crucial nature of MQC and evidence of NVU dysfunctions after stroke, we will discuss the molecular mechanisms of MQC, namely the alteration of mitochondrial dynamics (fission and fusion) and mitophagy after stroke. We highlight the regulation of MQC as a potential therapeutic target for both ischemic and hemorrhagic stroke.

## Mitochondrial Fission in Ischemic and Hemorrhagic Stroke

### Excessive Fission Aggravates Neurovascular Unit Dysfunctions

Mitochondrial fission is defined as a complex condition that divides single mitochondrion into two separate mitochondria (Ni et al., 2015). Previous work has established that excessive activation of mitochondrial fission is an underlying factor in after-stroke brain injury (Table 1). Excessive fission subsequently destroyed mtDNA integrity and decreased respiratory complexes (Wu et al., 2019). Mice with transient middle cerebral artery occlusion (tMCAO) that lacked A-kinase anchoring protein 1 (AKAP1, protein kinase A (PKA) scaffold) impaired the phosphorylation of Drp1 (fission-associated protein) at Ser637. Then, this led to overmuch fission, oxidative respiratory chain damage, Ca^2+^ dysregulation, and consequently deteriorative injury in ischemic brain issues (Flippo et al., 2018). AMP-activated protein kinase (AMPK) mediating the phosphorylation of AKAP1 caused the disassembly of PKA/AKAP1, which ultimately facilitates mitochondrial fragments (Zhang et al., 2017). In agreement, a few articles have reported that the maintenance of the phosphorylated state of Drp1 Ser637 *via* either the administration of Kaempferol (Wu et al., 2017) or the knockout of a neuron-specific Drp1 promotor [protein phosphatase 2A regulatory domain Bβ2 (Flippo et al., 2020)] can elongate the mitochondria and alleviate the damage in mice brain infarct area and primary neurons exposed to oxygen-glucose deprivation (OGD).

**TABLE 1:** Fission cellular pathways and their impacts on neurovascular unit (NVU) survival.

MQC	Major proteins	Disease model	Cellular pathways	Impacts on NVU
Fission	Drp1	Ischemia	Drp1 Ser637 dephosphorylation, caused by disturbance of the AMPK/AKAP1 pathway, leads to excessive activation of mitochondrial fission.	Increased fission damages oxidation respiratory chain, Ca2+ homeostasis, and consequently deteriorates injury in ischemic brain issue
		Ischemia	Maintenance of Drp1 Ser637 phosphorylation by Bβ2 knockout can elongate mitochondria.	Reduced fission alleviates damage in mouse brain infarct areas and primary neurons exposed to OGD.
		Ischemia	Phosphorylation of Drp1 at Ser616 and translocation by Atractylenolide III prevents extreme mitochondrial fission.	It moderates the CNS immune response by decreasing pro-inflammatory cytokines and increasing anti-inflammatory molecules (IL-10, CD206, and Arg-1, for instance) in the microglia of tMCAO rats
		Ischemia	A ketogenic diet is reported to prevent tMCAO-induced endoplasmic reticulum stress and mitochondrial cleavage.	Decreased stress alleviates activation of NLRP3 inflammasome and release of pro-inflammatory factors like IL-1β, IL-18, and caspase 1 from the mouse ischemic brain region and OGD/R SH-SY5Y cells.
		LPS stimulation	Excessive mitochondrial fission activates a cascade amplification of inflammation between microglia and astrocytes.	The fragmented mitochondria and inflammatory factors released from microglia trigger reactive astrocytes to transform into the pro-inflammatory A1 phenotype.
		ICH	Drp1 was over-activated, which inevitably contributed to a decrease in the anti-apoptosis associated protein, Bcl-2, and an increase in pro-apoptosis proteins (apoptosis-inducing factor, cytochrome c, caspase-3, etc.).	Dysregulated fission evokes neuronal apoptosis.
		Ischemia	Upregulated Mul1 intensifies fission by sumoylating Drp1 and ubiquitinating Mfn2.	Mul1 overexpression triggers cell apoptosis in ischemic brain tissues of rats.
		Hypoxia	Proper fission eliminates wasted mitochondrial segments, promotes signal transduction, boosts ATP production, and stabilizes mtDNA	Increasing mitochondrial fragments enhance the energy production of astrocytes and protect neurons from hypoxia damage.

Multiple evidence have advocated that the disruption of mitochondrial activities is prone to generating mitochondria-related damage-associated molecular patterns (DAMPs) and redundant ROS into the cytoplasm and an extracellular matrix, which are the major culprits in eliciting neuroinflammation (van Horssen et al., 2019; Liu et al., 2020). An experimental study using two-photon imaging in the neocortex of mice confirmed that an increment of mitochondrial fragmentation (fission) is obvious and reversible in cerebral ischemia injury (Kislin et al., 2017). Moreover, the expression of Drp1 was validated to increase in rat’s ipsilateral infarct area and BV-2 microglial cells subjected to OGD/reperfusion (OGD/R) (Rutkai et al., 2019; Hu et al., 2020). The inhibition of Drp1 phosphorylation at Ser616 and the translocation with Atractylenolide III prevent extreme mitochondrial fission, which subsequently moderates the CNS immune response by decreasing pro-inflammatory cytokines and increasing anti-inflammatory molecules (IL-10, CD206, and Arg-1, for instance) in the microglial cells of tMCAO rats (Zhou et al., 2019). In addition, a ketogenic diet was reported to prevent tMCAO-induced endoplasmic reticulum stress and mitochondrial cleavage. This restriction of fission restrained the activation of NACHT, LRR, and PYD domains-containing protein 3 (NLRP3) inflammasome, thereby reducing pro-inflammatory factors like IL-1β, IL-18, and caspase 1 in mice ischemic brain region and OGD/R SH-SY5Y cells (Guo et al., 2018; Toldo and Abbate, 2018).

Excessive mitochondrial fission was demonstrated to activate an amplification of the inflammation cascade between microglia and astrocytes. Park et al. (2013) have observed the phenomenon of increased mitochondrial fission in cultured microglia stimulated by lipopolysaccharides (LPSs). These mitochondrial fragments then regulated the microglia to be polarized into a pro-inflammatory phenotype (M1) for potentiating the expression of pro-inflammatory mediators (Park et al., 2013). The fragmented mitochondria and inflammatory factors released from microglia triggered reactive astrocytes to be transformed into a pro-inflammatory A1 phenotype that upregulated the expression of classic complements, i.e., C1r, C1s, C3, and C4 *in vitro* (Joshi et al., 2019; Liu et al., 2020). By highly expressing C3 that could bind with C3 receptors on microglia, A1 astrocytes exaggerated microglial inflammation and impaired microglial phagocytosis of myelin debris (Zheng et al., 2021). These results imply that the maintenance of mitochondrial homeostasis is beneficial to restore cerebral blood flow, dwindle infarct brain regions, alleviate cerebral edema, reduce cell apoptosis, and improve neurological outcomes.

Apart from inflammation, mitochondrial fission is also accompanied by apoptosis, indicating a cross-talk between apoptosis and mitochondrial dynamics. Mitochondrial fission is generally considered as an observed index of cellular apoptosis and is a crucial factor in apoptotic signaling pathways (Jeong and Seol, 2008; Maes et al., 2019). Drp1 was found to be overactivated in astrocytes surrounding hemotoma, which inevitably contributed to secondary brain injury after intracranial hemorrhage (ICH) in rodents (Wu et al., 2020; Zhang et al., 2021). Drp1 interference in ICH rats was shown to exert neuroprotection. While some articles suggest that these neuroprotective effects are attributed to the alleviation of inflammation, the downregulation of Drp1 can also suppress neuronal apoptosis following ICH and ischemic stroke. It was proven by the raised level of anti-apoptosis-associated protein B-cell lymphoma-2 (Bcl-2) and the reduced apoptosis-associated proteins (apoptosis-inducing factor, cytochrome c, caspase-3, etc.) (Xu et al., 2017; Zhang et al., 2021). Activated Bcl-2-associated-x protein (Bax) could be inserted into the outer mitochondrial membrane (OMM) to conduct oligomerization and finally increase OMM permeabilization (OMMP) (Zhao et al., 2014). Similarly, Mul1 was found to be upregulated in the ischemic tissues of rats in the tMCAO model, which intensified the fission by sumoylating Drp1 and ubiquitinating Mfn2 (Ren et al., 2019). The knockdown of Mul1 decreased a mitochondrial fragment while increasing energy generation, thus weakening caspase-3 activity, delaying apoptosis, and ameliorating the neurological deficit score (Ren et al., 2019). The application of photobiomodulation could reduce overwhelmed Drp1 GTPase activity and therefore restrain mitochondria-dependent caspase-3/9-apoptosome activities in the hippocampal CA1 regions of cardiac-arrest-induced global cerebral ischemic rats (Wang R. et al., 2019). Being independent of Drp1, mitochondrial division dynamin can promote Bax-associated cytochrome c release and apoptosis in the cell strain RDY84, which can be attenuated by the administration of a mitochondrial division inhibitor (mdivi-1) (Cassidy-Stone et al., 2008). Interactionally, the pro-apoptotic Bcl-2 subfamily, including Bax and BH3 homolog agonist killer (Bak), was capable of recruiting Drp1 to the fission site and actuating its fission activity (D’Orsi et al., 2017).

### Fission Acts as a Neuroprotective Factor

Although lots of research as mentioned above considers mitochondrial fission as a harmful process in stroke-induced brain injury, some evidence suggests its neural protective effect by eliminating the wasted mitochondrial segments, promoting signal transduction, boosting ATP production, and stabilizing mtDNA. Research on HEK293 and HeLa cells has found that the inhibition of Drp1-induced fission impaired the selectivity of mitophagy, thus enhancing its rate of elimination of even healthy mitochondria *via* the unchecked PINK1/Parkin pathway (Burman et al., 2017). After selective overexpression of uncoupling protein-2 (UCP2) in the corpus striatum of stroke-prone spontaneously hypertensive (SHRSP) rats, Busceti et al. (2020) found the upregulation of OPA1 and Fis1, which respectively amplified mitochondrial fusion and fission in the striatum tissue. Although the causality was not explicit, this experiment implied that UCP2 exerted neuroprotection by driving mitochondrial fusion and fission. A growing body of literature has investigated that asymmetric division of the mitochondrial body could separate the functional segment from the dysfunctional one. The dysfunctional one is then eliminated by mitophagy (Zorov et al., 2019). These physiological processes can confine the flawed mitochondria to precise clearance and maintain the integrity of the mitochondrial structure. Fission and mitophagy damages may give rise to the accumulation of abnormal mitochondrial elements that initiate various pathologies, jeopardize the whole mitochondrial network, and even kill the cell (Ma et al., 2020). Though mitochondrial fission is conventionally thought to be detrimental to cellular energy production and homeostasis, Horn et al. (2020) have identified that mitochondrial fragments enable mouse embryonic fibroblasts (MEFs) and HeLa cells to prime protective signal pathways on focal cell membrane damage. This process is indispensable to repair and regenerate the cells damaged by laser assay (Horn et al., 2020). Furthermore, increasing mitochondrial fragments seemed to amplify the energy production in primary astrocytes during hypoxia *in vitro* (Quintana et al., 2019). Mice that postnatally experienced Drp1 knockdown were shown to suffer from a deficit in bioenergetics and synaptic transmission (Shields et al., 2015). In addition, fission and other MQC mechanisms are involved in maintaining mtDNA integrity through the removal of mutational and dysfunctional mtDNA (Mishra and Chan, 2014). Irregularity in these processes could result in mitochondrial disorder and neuronal damage.

The regulations of mitochondrial fission that contain various molecules, organelles, and signaling pathways can diversely influence several cellular functions, which require more research to explore the mechanism of its interaction with stroke progression. It remains controversial whether restraining fission is beneficial to protect cell viability after stroke. As mitochondrial fission appears to be a double-edged sword, there is a possible delicate balance between the physical compensation and the pathological initiation. Further clinical research must be conducted to validate the therapeutic effect of mitochondrial fission administration because of the complex MQC network and a wide heterogeneity of individual stroke pathology (for instance, location, duration, severity, and fundamental conditions).

## The Axes of Mitochondrial Fission

**FIGURE 2:**
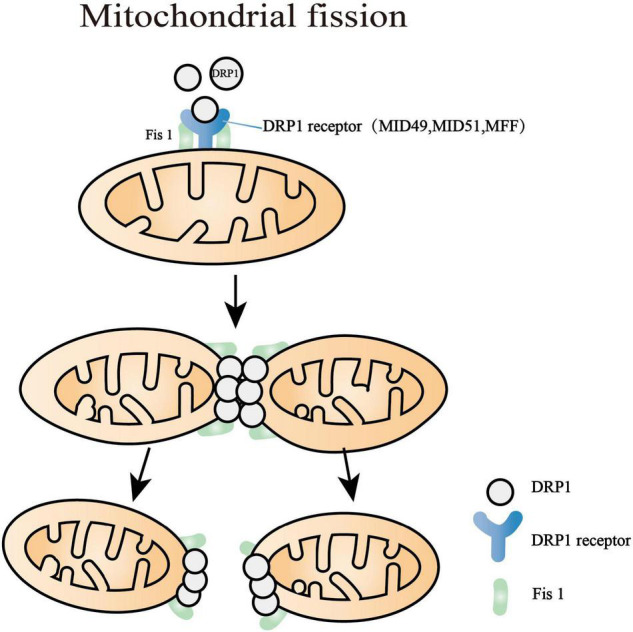
Mitochondrial fission processes. Dynamin-related protein 1 (Drp1) is the major motivator of fission that is regulated by diverse protein kinases and posttranslational modifications. The major targets of Drp1 are mitochondrial fission factor (MFF), mitochondrial dynamics proteins of 49 and 51 kDa (MID49, MID51), and fission protein 1 (Fis1). After the polymerization of Drp1 with MID49 and MID51, the oligomerized Drp1 filament curled into a constricted ring with a 16-nm internal diameter. The last step of the mitochondrial division is myosin II recruitment, which motivates deformations of the actin network and completes further mitochondrial constriction.

Several dynamin-related GTPase proteins control the mitochondrial dynamic transitions (Tilokani et al., 2018), among which Drp1 is the major motivator in the progression of mitochondrial fission (Xie et al., 2015; Figure 2). Drp1 is a cytosolic molecule that can be recruited to mitochondria and then cleave the mitochondrial membrane. It is regulated by diverse protein kinases and posttranslational modifications. Drp1 dephosphorylation of serine 637 by Ca2 + -dependent phosphatase calcineurin enhances GTPase and elicits mitochondrial fission (Cereghetti et al., 2008), whereas the phosphorylation of the same point using PKA results in mitochondrial elongation (Cribbs and Strack, 2007). Besides, another phosphorylated site of Drp1 on serine 616 using PINK or PKCδ can drive it to be translocated to OMM and hence promote mitochondrial fission (Giacomello et al., 2020; Han et al., 2020). Drp1 phosphorylation at serine 585 using Cdk1/Cyclin B increases its GTPase activity and facilitates mitochondrial fragment during mitosis (Taguchi et al., 2007). Apart from phosphorylated and dephosphorylated modifications, the activity of Drp1 is also regulated by mitochondrial E3 ubiquitin ligase (Mul), a multifunctional OMM protein, which elicits both sumoylation and ubiquitylation of Drp1 (Karbowski et al., 2007; Peng et al., 2016). Mul was regarded as a stabilizer of Drp1, namely promoting its subcellular translocation and steadying endoplasmic reticulum-mitochondrial contact sites (Prudent et al., 2015). The shRNA-mediated loss of Mul led to markedly interconnected and lengthened mitochondria, which promoted the senescence of HeLa and Chang cells (Park et al., 2010). In contrast, Mul knockout in human colorectal carcinoma cells resulted in mitochondrial fragmentation (Xu et al., 2016). A reasonable explanation might be that Mul had another function of mediating ubiquitination and degradation of mitochondrial dynamic proteins of 49 (MiD49), which reverses abnormal recruitment of Drp1 and mitochondrial fission. This inconsistency is probably associated with different cell types, discrepant pathophysiological progress, and changing mitochondrial bioenergetics. Thus, further studies both *in vivo* and *in vitro* are needed to precisely explain the molecular regulation of mitochondrial fission and its equilibrium point, which may be novel therapeutic targets.

Multiple pieces of evidence have demonstrated that the major targets of Drp1 are mitochondrial fission factor (MFF), mitochondrial dynamics proteins of 49 and 51 kDa (MID49 and MID51), and mitochondrial fission protein 1 (Fis1) (Losón et al., 2013; Liu and Chan, 2015; Atkins et al., 2016; Otera et al., 2016). These receptors are located at the OMM and play an essential role in recruiting and activating Drp1 (Kalia et al., 2018). After the polymerization of Drp1 with MID49 and MID51, the oligomerized Drp1 filament curled into a constricted ring with a 16-nm internal diameter accompanied by GTP hydrolysis (Kalia et al., 2018). Overall, Drp1 must exert abundant conformational conversion to mediate mitochondrial fission. The last progress of the mitochondria division is myosin II recruitment, which motivates deformations of the actin network and thus completes further mitochondrial constriction (Yang and Svitkina, 2019).

## Mitochondrial Fusion in Stroke

Mitochondrial fusion is a complicated process that requires the coordinated fusion of the OMM and the inner mitochondrial membrane (IMM) from adjacent mitochondria. Data from various sources have shown that, after stroke, the expression of fusion-associated proteins, Mfns and OPA1, decreased and affected the mitochondrial fusion process (Table 2). The reduction of Mfn2 in OGD neurons or middle cerebral artery occlusion (MCAO) rats induces Ca2 + overload and Bax translocation to mitochondria. It subsequently caused cytochrome c release, OMMP, and neuron apoptosis (Martorell-Riera et al., 2014). In turn, mitochondrial Bax translocation during apoptosis could block mitochondrial fusion in primary human myocytes (Karbowski et al., 2004), indicating that apoptosis activation and fusion inhibition might be a process of mutual promotion. The overexpression of Mfn2 reduces phosphorylated extracellular signal-regulated kinase (p-ERK) and alleviates p-ERK-associated apoptosis in hypoxia-treated neurons *in vitro* and in the ischemic area of permanent MCAO mice (Peng et al., 2015). OPA1 has shown a significant decrease in rat primary cortical neurons subjected to OGD (Lai et al., 2020), which is possibly owing to pathological stimulation of ATP-independent metalloprotease (OMA1) (Wai et al., 2015).

**TABLE 2:** Fusion cellular pathways and their impacts on NVU survival.

MQC	Major proteins	Disease model	Cellular pathways	Impacts on NVU
Fusion	Mfn1 Mfn2 OPA	Ischemia	Reduction of Mfn1 and Mfn2 in OGD neurons and MCAO rats induces Ca2+ overload and Bax translocation to mitochondria.	Impaired fusion causes cytochrome c release, OMM permeabilization, and neuronal apoptosis.
		Ischemia	Mfn2 overexpression inhibits the pro-apoptotic protein p-ERK.	Mfn2 protects neurons from ischemic injury.
		Ischemia	Mfn2 can increase the Bcl-2/Bax ratio and decrease the level of cleaved-caspase-3 through the ERK-Bcl-2/Bax pathway.	Mfn2 plays an anti-apoptotic role in hypoxia-induced neurons and in the ischemic areas of permanent MCAO mice.
		OGD	OPA1 is inhibited by OMA1 in rat primary cortical neurons subjected to OGD.	OMA1 causes impairment of fusion and aggravates neuronal death by downregulating OPA1.
		Ischemia	OPA1 activated by melatonin can activate the Yap-Hippo pathway and reverse mitochondrial fusion.	Fusion recovery ameliorates neuronal function and improves energy metabolism.
		SHRSP	UCP2 promotes the expression of OPA1 and Fis1, which respectively amplifies mitochondrial fusion and fission in the striatum tissue.	UCP2 protects neurons by promoting mitochondrial fusion and fission.

The revival of fusion has been found to reduce mitochondrial oxidative stress and apoptosis in stroke. The overexpression of fusion-associated proteins, such as Mfns and OPA1, in hypoxia-induced neurons and also in the ischemic area of permanent MCAO mice was documented to alleviate mitochondrial dysfunction and cellular apoptosis. Beyond promoting mitochondrial fusion, the revival of Mfn2 was also shown to increase the Bcl-2/Bax ratio and decrease the cleaved caspase-3 level through the ERK-Bcl-2/Bax pathway. This phenomenon represents Mfn2 as a potentially anti-apoptotic factor after ischemic stroke (Peng et al., 2015). Bax-interacting factor 1 (Bif-1) was found to be downregulated in neurons from the penumbra of ischemic stroke mice, which was thought to be associated with fragmented mitochondria and exaggerated apoptosis (Wang et al., 2014). The overexpression of Bif-1b/c (the neuron-specific isoforms) promoted mitochondrial elongation, maintained membrane potential, ultimately attenuated apoptosis, and improved neural viability (Wang et al., 2014). It was observed that the intake of a granulocyte-colony stimulating factor (GCSF) in the striatum, cortex parts, and hippocampus of globally ischemic stroke mice, resulted in the upregulation of OPA1 accompanied by an increase of the anti-apoptotic molecule Bcl-2 and a decrease of pro-apoptotic factors Bax, Bak, and p53-upregulated modulator of apoptosis (PUMA) (Modi et al., 2020). Level restoration of effective OPA1 was demonstrated to protect the postischemic brain from mitochondrial incompleteness and defective bioenergetics. Then, this inhibited the apoptosis of rat primary cortical neurons subjected to OGD and the ischemic region of tMCAO rats (Lai et al., 2020). The expression, stabilization, and activation of OPA1 are associated with AMPK, ERK, and Yap pathways (Xin et al., 2020). Melatonin-activated OPA1 was shown to activate the Yap–Hippo pathway, to reverse mitochondrial fusion, and to reduce cerebral reperfusion stress in the ischemic area of tMCAO mice and N2a neuroblastoma cells exposed to hypoxia-reoxygenation *in vitro*, as evidenced by the ameliorative neural function and improved energy metabolism (Wei et al., 2019).

## The Axes of Mitochondrial Fusion

**FIGURE 3:**
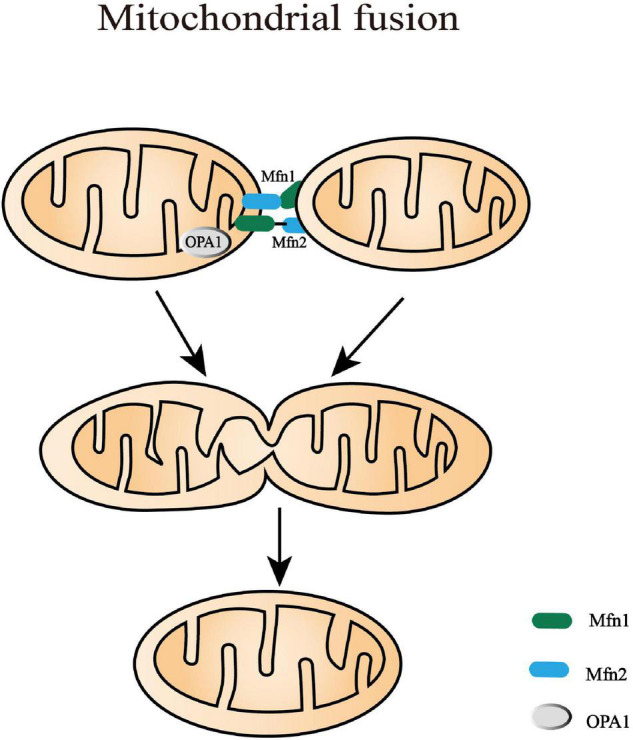
Mitochondrial fusion processes. Mitochondrial fusion is a complicated process that needs to coordinate the fusion of outer mitochondrial membrane (OMM) and inner mitochondrial membrane (IMM) from adjacent mitochondria. In mammals, OMM fusion is mediated by mitofusin 1 and mitofusin 2 (Mfn1 and Mfn2) that prime fusion as they form oligomers. Following the decreasing distance of the two OMM, GTP hydrolysis triggers conformational changes of the Mfn oligomers and subsequently mediates local OMM fusion. The IMM fusion that occurs following OMM fusion is induced by a dynamin-like GTPase, optic atrophy type 1 (OPA1). It is alternatively processed into eight multifunctional isoforms by presenilin-associated rhomboid-like protein (PARL), AAA protease, and OMA1 to precisely regulate fusion activity.

In mammals, OMM fusion is mediated by mitofusin 1 (Mfn1) and Mfn2, both of which are transmembrane proteins containing a GTPase domain (Pernas and Scorrano, 2016; Figure 3). Mfn1 and Mfn2 appear to play a distinct but also repetitive role in the fusion process. Mfn1 has higher activity in tethering adjacent mitochondrial membrane while Mfn2 can couple OMM to the endoplasmic reticulum (Chen et al., 2003; McLelland and Fon, 2018). In addition, Mfn2 rather than Mfn1 contributes to the adaptation of stress and pro-inflammatory activation in macrophages (Tur et al., 2020). However, the knockdown of either Mfn1 or Mfn2 results in an obvious mitochondrial fragment and decreased oxidative phosphorylation function, while the overexpression of either of them is sufficient to restore fusion (Tilokani et al., 2018). Mfn1 and Mfn2 prime fusion as they form oligomers on the same mitochondrion or between adjacent mitochondria to extend the contact surface by tethering and docking the two OMMs (El-Hattab et al., 2018). Following the decreasing distance of the two OMMs, GTP hydrolysis triggers conformational changes of the Mfn oligomers and subsequently mediates local OMM fusion (Brandt et al., 2016).

The IMM fusion that occurs following OMM fusion is induced by a dynamin-like GTPase, OPA1. OPA1 is alternatively processed into eight multifunctional isoforms to precisely regulate fusion activity by presenilin-associated rhomboid-like protein (PARL), AAA protease, and OMA1 (or named MPRP1) (MacVicar and Langer, 2016). These proteases vary in time/tissue-specific expression and splice variants, which can cleave OPA1 into long and short isoforms (L-OPA1 and S-OPA1). The balance between L-OPA1 and S-OPA1 plays an important role in maintaining the cristae structure, mtDNA stability, energetic competency, and providing protection against cellular stress (Lee et al., 2017, 2020). Moreover, cardiolipin, a mitochondria-specific phospholipid in IMM, is required for the biogenesis, translocation, and oligomerization of OPA1 (Kameoka et al., 2018). In liposomes without enough cardiolipin, OPA1 can only tether them together. Only when the liposomes contain abundant cardiolipin domains, can OPA1 become fusogenic and promote the fusion of liposomes (Liu and Chan, 2017). Mechanically, L-OPA1 was confirmed to mediate the hemifusion of membranes and low-level release of content. L-OPA1 can also be further cleaved into S-OPA1 that coordinates with L-OPA1 to efficiently open the membrane pore (Ge et al., 2020). However, excessive S-OPA1, cleaved by OMA1, inhibits fusion and promotes the fission of the impaired mitochondria with decreased membrane potential or ATP level (MacVicar and Langer, 2016). This survival mechanism can prevent the dysfunctional mitochondria from mixing into the whole mitochondrial network, which is beneficial to quality control.

## Promoting the Balance Between Fission and Fusion Attenuates Neurovascular Unit Injury

Previous research has indicated that the balance between mitochondrial fission and fusion dynamically impacts the shape and homeostasis of the mitochondria (Murata et al., 2020). These complex changes in the mitochondrial structure are intimately linked to their functional versatility (Dong et al., 2021). Mitochondrial fusion and fission can be regulated by selectively blocking or promoting either of these processes. Namely, fission is increased when fusion is blocked or fusion is augmented when fission is inhibited (Adebayo et al., 2021).

The balance between mitochondrial fission and fusion modulates neuroinflammation, neural apoptosis, cell metabolism, cell cycle, BBB, etc. (Scott and Youle, 2010; Horbay and Bilyy, 2016). The reduction of ShcA, a ROS regulatory protein, could reverse the increased level of Drp1, Fis1/Mfn1 (the fission and fusion protein, respectively), and PINK1, which indicated that the mitochondrial dynamic balance tilted from fission to fusion (Hwang et al., 2020). This moderate equilibrium achieved by diminishing the expression of ShcA contributed to reduced infarct volume and mitigatory neurological deficits in Rose Bengal photothrombotic mice (Hwang et al., 2020). Consistently, in hyperglycemia-exacerbated MCAO models, reducing Drp1 and promoting Mfn2 also made sense in elevating mitochondrial membrane potential and suppressing ROS production in the ischemic brain area (Li et al., 2017). However, in-depth mechanistic studies are needed to identify the mechanisms involved in mitochondrial fusion and fission transition and the proper balance point in neuroprotection.

## Mitophagy in Stroke

Mitophagy is a word originating from mitochondrial autophagy that plays an indispensable role in regulating mitochondrial degradation. It belongs to macroautophagy, with the process of autophagosome formation and degradation in lysosomes (Swerdlow and Wilkins, 2020). Mitophagy is a complex process where multiple proteins are involved, like PINK1, Parkin, BNIP3, NIX, autophagy-related proteins (Atg), LC3, FUNDC1, etc.

### Activity of Mitophagy in Stroke

Several studies have suggested that mitophagy is suppressed after stroke (Table 3; Di et al., 2015; Wang H. et al., 2019). Agents promoting mitophagy could be potential therapeutic candidates to protect mitochondrial function and ameliorate neural injury after stroke. Deng et al. (2021) showed that decreased PINK1 expression and mislocated Parkin existed both in the peripheral-hematoma brain tissues of ICH rats and in the oxygen hemoglobin-treated primary neurons. The elevation of mitochondrial membrane potential using methylene blue or electroacupuncture augmented mitophagy and subsequently attenuated neural injury in the ischemic area of tMCAO rats and OGD neuron-like PC12 cells (Di et al., 2015; Wang H. et al., 2019). Nicotinamide phosphoribosyltransferase (Nampt) knockdown inhibited autophagy, contributing to aggravated apoptosis and necrosis in the cultured cortical neurons subjected to OGD and the ischemic area of tMCAO rats (Wang et al., 2012). The block of Nampt can enhance the phosphorylation of mTOR and S6K1, which abolished the expression of autophagic proteins LC3 and Atg6 (Wang et al., 2012).

**TABLE 3:** Mitophagy cellular pathways and their impacts on NVU survival.

MQC	Major proteins	Disease model	Cellular pathways	Impacts on NVU
Mitophagy	PINk1 Parkin BNIP3 NIX Atg LC3 Nrf2 OPTN	ICH	Decreased PINK1 and mislocated Parkin are found both in the peripheral-hematoma brain tissues of ICH rats and oxygen hemoglobin treated primary neurons.	Insufficient mitophagy is associated with neuronal death.
		Ischemia	The levels of LC3II/LC3I, PINK1, Parkin, Beclin1, and LC3-COX4 are significantly elevated in ischemic hemisphere brain tissues.	Excessive mitophagy may be linked to oxidative stress, as evidenced by an increase in TNF- and IL-1 levels.
		ICH	Nrf2/OPTN-mediated mitophagy reduces NLRP3 inflammasome and improves mitochondrial function.	Proper mitophagy protects hippocampal neurons and reduces brain edema.
		Ischemia	Nampt inhibition increases mTOR and S6K1 phosphorylation, which inhibits the expression of autophagic proteins LC3 and Atg6.	Knockdown of Nampt inhibits autophagy and aggravates neuronal apoptosis and necrosis in ischemic areas of tMCAO rats.
		SHRSP	Impaired mitophagy caused by Ndufc2 gene downregulation leads to deficient removal of the damaged mitochondria and the accumulation of misfolded proteins.	Autophagy inhibition throughout the brain may result in endothelial cell injury and increased stroke morbidity.
		SAH	Mitophagy promoted by Mitoquinone can mitigate mitochondrial oxidative stress and preserve mitochondrial integrity *via* Nrf2/Keap1 and PINK1/Parkin pathways.	Enhanced mitophagy counteracts apoptosis by eliminating depolarized mitochondria in neurons.
		Neonatal I/H	Expression of BNIP3 increased in a delayed manner.	Overactivation of mitophagy contributes to deteriorative neural losses.
		Ischemia	Increased mitophagy reduces the production of inflammatory factors in microglia and astrocytes.	The neuroprotective effect of mitophagy is achieved *via* alleviating inflammatory responses in glia.
		Astrocyte H/I	The enhanced mitophagy and recovered ATP production during reoxygenation contribute to a reduction in mitochondrial quantity and loss of astrocytic extensions.	The confined astrocytic extension might ameliorate glial scar formation and consequently reverse its inhibition of axonal growth and regeneration.
		SAH	LC3-II, Atg5, Parkin, and PINK1 are increased at 24 h after SAH in ipsilateral basal cortical samples nearing the blood clots.	Further activation of mitophagy at the early phase of SAH promotes dysfunctional mitochondria elimination, ROS reduction, and inflammation alleviation.
		SAH with DCI	A significant increase in the mRNA expression of mitophagy markers (DAPK1, BNIP3L, and PINK1) is found in SAH patients with DCI.	Enhanced mitophagy, concomitant with remarkable mitochondrial dysfunction, might be involved in DCI pathogenesis.
		Ischemia	Inhibition of Drp1 counteracts selective mitophagy, causing the accumulation of the damaged mitochondria.	Selective clearance of the damaged mitochondria can promote cellular survival, while non-selective mitophagy leads to neural injury.

While reduced mitophagy has been intensively reported, some articles suggest that mitophagy is activated in stroke models with evidence of typical morphology, mitophagy makers, and mitochondrial mass. This phenomenon was found in MCAO models. The levels of LC3II/LC3I, PINK1, Parkin, Beclin1, LC3-COX4 copositive puncta were significantly elevated in ischemic hemisphere brain tissues (Li Q. et al., 2014; Li et al., 2018; Zuo et al., 2019; He et al., 2021). Research using long time-lapse imaging has discovered that Parkin was selectively recruited to the damaged mitochondria in the somatodendritic regions of neurons during CCCP exposure (Cai et al., 2012). It subsequently enhanced the translocation of the mitochondria to lysosomes for autophagy. Through a mitochondria-targeting two-photon ratiometric probe, enhanced mitophagy was demonstrated at 0, 3, 6, and 12 h after ODG/R in PC12 cells (Cheng F. et al., 2021). Increased mitophagy may be related to oxidative stress. Anti-inflammatory treatment can partially inhibit mitophagy (Cheng F. et al., 2021). Under physiological conditions, the autophagy adaptors BNIP3 and Beclin1 bind with Bcl-2 at the BH3 domain, which decreases mitophagy activity. In the cortex, striatum, and hippocampus of tMCAO mice and OGD-disposed hippocampal neuronal HT22 cells, uncoupling of Beclin-1/Bcl-2 complexes induced mitophagy, which led to reduced infarct size and brain edema (Chen et al., 2020). Nur77 is an orphan nuclear receptor that is dupregulated during inflammation and then in turn restricts inflammation development (Lith and de Vries, 2021). In MCAO rats and ODG/R human SH-SY5Y cells, Ping et al. (2021) have reported that increased mitophagy was induced by the translocation of Nur77 from the nucleus to mitochondria. Heat shock protein B8 (HSPB8) upregulated in tMCAO was shown to reinforce mitophagy in CA1 sector of the hippocampus (Li et al., 2018). The overexpression of HSPB8 further promoted mitophagy and therefore attenuated cell apoptosis after ischemic stroke. Meanwhile, this neuroprotective effect of HSPB8 could be compromised by inhibiting mitophagy (Li et al., 2018). In conclusion, it is likely that mitophagy is transiently induced after stroke and serves as a spontaneously neuroprotective response.

### Mitophagy Acts as a Neuroprotective Factor

Mitophagy eliminates the damaged mitochondria and mitigates inflammation after stroke. It was shown that mitochondrial stress was booming after exhaustive exercise of PINK1^–/–^ mice or mtDNA mutation of Parkin^–/–^ mice. These mitophagy-deficient mice release extensive DAMPs, which remarkably activate a strong inflammatory response in the heart tissue of mice subjected to exhaustive exercise (Sliter et al., 2018). A study on SHRSP rats has provided evidence that autophagy inhibition in the whole brain may lead to increased morbidity from stroke (Forte et al., 2020). This may be associated with the deficient removal of the damaged mitochondria and the accumulation of misfolded proteins (Forte et al., 2020). Mechanically, the impaired mitophagy was likely the consequence of Ndufc2 gene downregulation in the SHRSP brain, contributing to NAD^+^ reduction (Forte et al., 2020). The reduction of NAD^+^ weakens cellular antioxidation and energy metabolism, leading to apoptosis and necroptosis in OGD/R-treated primary cultured neurons and in the ipsilateral areas of MCAO/R mice (Huang et al., 2018).

Recently, the central roles of mitophagy in mitigating neuronal apoptosis and increasing neural function have also been reported. In the ischemic cerebral cortex and the OGD hippocampal neuron cell line HT22, ischemia-reperfusion injury-induced oxidative stress and increased the production of caspase3 and Bax while decreasing the expression of the antiapoptotic molecule Bcl-2. These effects can be reversed by tissue-type plasminogen activator (tPA) treatment (Cai et al., 2021). tPA inhibited apoptosis and maintained the mitochondrial function through upregulating FUNDC1. FUNDC1 is an OMM protein that recruits LC3 to trigger mitophagy in mammals (Chen et al., 2016). The knockdown of FUNDC1 destroyed LC3/mitochondrial colocalization, hence lessening the neuroprotective effects of tPA-mediated mitophagy (Cai et al., 2021). A similar conclusion was acquired from a study on subarachnoid hemorrhage (SAH) in rats. Mitophagy promoted by Mitoquinone was able to mitigate mitochondrial oxidative stress, preserve mitochondrial integrity, and ultimately counteract apoptosis *via* Nrf2/Keap1 and PINK1/Parkin/LC3II pathways in the nucleus and mitochondria, respectively (Zhang et al., 2019). Moreover, enhanced mitophagy was suggested to raise neural resistance to ischemic stroke and lengthen the time window of thrombolytic therapy in MCAO-treated mice (Shen et al., 2017). This dynamic process increases the efficiency of eliminating the depolarized mitochondria in mature cortical neurons, which makes sense in attenuating neural damage.

### Excessive Mitophagy Aggravates Neural Injury

Excessive mitophagy is accompanied by multitudinous mitochondrial damage, degradation, and metabolic dysfunction. Using a neonatal Ischemia/Hypoxia (I/H) mouse model, Shi et al. (2014) observed that the expression of BNIP3 increased in a delayed manner (increasingly elevated in 1, 3, and 7 days), contributing to deteriorative neural loss. The knockdown of BINP3 obviously mitigated mitophagy and neuronal apoptosis in ischemic and hypoxic insults (Shi et al., 2014). Consistently, it was found that in response to cerebral ischemia-reperfusion injury, small nucleolar RNA host gene 14 was upregulated in mouse hippocampal neurons, which excessively stimulated mitophagy and induced cell apoptosis (Deng et al., 2020). Research on similar tMCAO (occlusion for 2 h and reperfusion for 22 h) rats reported that PINK1, Parkin, and LC3-II were increased in ischemic brain tissues (Zhang Y. et al., 2020). Rehmapicroside treatment prevented the accumulation of mitophagy-associated proteins on the mitochondria and simultaneously improved neurological deficit scores (Zhang Y. et al., 2020). Increased mitochondrial dysfunction and mitophagy also occurred in H_2_O_2_-treated primary cortical neuronal cells and at 24 h after Rose Bengal photothrombosis in cortical infarct mice (Hwang et al., 2020). ROS alleviation partially avoided mitochondrial damages and autophagy, which protected neurons against photothrombotic cerebral infarction (Hwang et al., 2020). A similar phenomenon was obtained from the severe ischemic model (Wang R. et al., 2019) – global cerebral ischemia (GCI) with bilateral common carotid arteries and vertebral artery occlusion. Wang R. et al. (2019) described that hippocampus CA1 neurons displayed marked nuclear membrane distortion, hyperchromasia, and fragmented mitochondria with broken cristae and membrane depolarization at 3 days after GCI. Additionally, the impaired mitochondrial network erupted numerous mitophagy, followed by the deficiency of cytochrome c oxidase and ATP contents and the increase of CA1 neuron apoptosis. Based on the abovementioned data, it seems likely that excessive mitophagy is not only a cause leading to the impaired mitochondrial network but also a result induced by the severe stroke.

## Mitophagy: Upregulated or Downregulated? Pro-Survival or Pro-Death?

It still remains controversial about the activity of mitophagy changing after stroke, and whether these changes are protective or detrimental. On one hand, mitophagy allows cells to clear the damaged mitochondria, which is beneficial to cellular component recycling and cellular homeostasis. On the other hand, excessive clearance of essential mitochondria can damage energy generation and mitochondria-associated signal pathways. The complex role of mitophagy might be related to the variant NVU cells, different time courses of stroke, dissimilar severity, and diverse selectivity, which will be discussed below.

### Cell Type

The activation of mitophagy after stroke is different in variant NVU cells like neurons, microglia, astrocytes, endothelia, etc. (Lan et al., 2018). Referring to their unique biological characteristics, the mitophagy-induced downstream pathways might play distinct roles in the prognosis of stroke. Evidence provided by Cai et al. (2012) has confirmed several specific features of Parkin-mediated mitophagy in cortical neurons. Firstly, Parkin was selectively recruited to dysfunctional mitochondria, which occurred more slowly than non-neurocytes. Secondly, Parkin translocation is restricted to somatodendritic regions of a small fraction of neurons where mature lysosomes are abundant. These spatial characteristics allow neurons to efficiently clear the depolarized mitochondria and maintain cellular vitality (Cai et al., 2012). Research on the mouse hippocampal neuron cell (HT22) OGD model has revealed that the activation of mitophagy by inducing FUNDC1 phosphorylation contributed to less neuronal apoptosis (Cai et al., 2021).

Mitophagy has been shown to play a similar protective role in endothelial cells (Forte et al., 2020). Endothelial progenitor cells (EPCs), isolated from the peripheral venous of human subjects with an NDUFC2 variant, exhibit mitophagy impairment and senescence aggravation when cultured at a high salt level (Zuo et al., 2019). Pharmacological activation of mitophagy restores EPCs’ mitophagy and reduces stroke occurrence in SHRSP rats.

As for microglia, the neuroprotective effect of mitophagy was achieved *via* alleviating the inflammatory responses that were induced by an ischemic stroke in mouse (Han et al., 2021). Though little research on stroke explored the function of microglial mitophagy, the anti-inflammatory role of microglial mitophagy has been reported in several diseases, including stress-induced hypertension (Zhang S. et al., 2020), glaucomatous neurodegeneration (Jassim et al., 2021), Alzheimer’s disease (Agrawal and Jha, 2020), Parkinson’s disease (Ahmed et al., 2021), etc. Meanwhile, the underlying mechanisms of microglial mitophagy in anti-inflammation are unclear, requiring further elucidation.

In astrocytes, mitophagy was shown to be upregulated after ischemic stroke *in vivo* and *in vitro* (Cao et al., 2021). Further activation of mitophagy limited astrocyte polarization to a pro-inflammatory subtype, thereby reducing the production of inflammatory cytokines and neurotoxins (Cao et al., 2021). In another hypoxia/reoxygenation model of primary astrocytes *in vitro* (Quintana et al., 2019), 24.9 and 43.7% of mitochondria, respectively, undergo mitophagy at 3 h after hypoxia and 10 h of reoxygenation, comparing to 26.4% of mitochondria under normoxic conditions. In addition, the authors have also observed a reduction of astrocytic ATP during hypoxia, but a restoration of ATP content after reoxygenation (Quintana et al., 2019). Enhanced mitophagy and the recovery of ATP production during reoxygenation contribute to decreased mitochondrial quantity and limited astrocytic extensions. This confined astrocytic extension might ameliorate glial scar formation, and consequently reverse its inhibition of axonal growth and regeneration (Liu and Chopp, 2016; Okada et al., 2018).

### Time Course

As stroke develops, the activity of mitophagy keeps changing, which indicates its intricate impact on secondary brain injury. We proposed that mitophagy activated at the acute stage of stroke might be neuroprotective to eliminate the damaged mitochondria, but persistent or immoderate mitophagy in the subacute and chronic stages could be harmful due to enormous mitochondrial degradation.

Mitophagy upregulated by acidic postconditioning in the early stage of ischemic stroke (within 6 h) could render the brain tissue resistant to ischemia in mice (Shen et al., 2017). This enhanced mitophagy may facilitate the clearance of the damaged mitochondria, subsequently eliminating the ROS burden and retarding disease promotion. Intervening mitophagy at the different stages of stroke could result in contrasting effects. Lan et al. (2018) have observed that the levels of mitophagy markers on neurons increased at 6 h after tMCAO, and then reached their peak at 24 h. The severe cellular insult at 24 h excessively motivated mitophagy, leading to the deficit of mitochondrial quality and energy generation (Anzell et al., 2018; Lan et al., 2018).

However, in different stroke models, the concrete time point dividing acute and chronic phases is different. Research on SAH rats has shown that both autophagy proteins (LC3-II and Atg5) and mitophagy markers (Parkin and PINK1) were increased at 24 h after SAH in ipsilateral basal cortical samples nearing the hematoma (Cao et al., 2017). Further activation of mitophagy by melatonin at 24 h after SAH promoted the fragmented mitochondrial elimination, concomitant with a reduction of ROS generation and inflammatory cytokine release (Cao et al., 2017). Increased mitophagy at 48 h after SAH in rats’ cerebrums also played a significant role in attenuating neuronal apoptosis and necrosis (Li J. et al., 2014). In addition, Cheng Y. et al. (2021) have reported that Nrf2/OPTN-mediated mitophagy, at 3 days after ICH in mice, inhibited NLRP3 inflammasome and improved the mitochondrial function in perihematomal brain tissues.

### Disease Severity

Another possible cause might be the different severity of stroke derived from the varied protocol of animal model establishment. Excessive mitophagy could be attributed to prolonged brain hypoperfusion, massive cerebral infarction, cellular stress after reperfusion, etc. These severe brain injuries experienced nutrient deprivation, DNA destruction, oxidative stress, and mitochondrial depolarization, which played a role in motivating hyperactive and protracted mitophagy. In rat permanent focal ischemia, Baek et al. (2014) have reported that Drp1 and Parkin increasingly recruited to the mitochondria, which resulted in a release of cytotoxic substances, cytochrome C, and apoptosis-associated factors in the ischemic area of pMCAO rats and primary cortical neurons treated by NMDA. Carnosine treatment attenuated ischemic injury in the ipsilateral hemisphere by decreasing mitophagy (Baek et al., 2014). This phenomenon implies that enormous mitochondrial damage and degradation occur in severe stroke models, leading to the deficiency of the mitochondrial network and impaired energy production. A similar effect was reported in GCI following cardiac arrest. Excessive mitochondrial fission and mitophagy contribute to massive hippocampal CA1 neuron apoptosis as evidenced by increasing mitochondrial oxidation, fragmented structure, and disintegrated cristae (Wang R. et al., 2019). On the contrary, cellular mitophagy was considered to play a pro-survival role in preconditioned tMCAO rats that experienced 30 min of tMCAO before 100 min of tMCAO (Matic et al., 2016). The extent of brain injury in this preconditioned tMCAO model might be moderate, thus the optimal mitophagy as a defensive reaction maintains the MQC homeostasis and increases the mitochondrial resistance to reoxygenation and membrane depolarization. Recently, Youn et al. (2021) have compared the morphological changes and autophagy of mitochondria in cerebrospinal fluid cells between patients with and without delayed cerebral ischemia (DCI) SAH. They found a significant increase in mRNA expression of mitophagy markers (DAPK1, BNIP3L, and PINK1) in patients with DCI SAH. This enhanced mitophagy, concomitant with remarkable mitochondrial dysfunction, might be involved in DCI pathogenesis (Youn et al., 2021).

### Selective Mitophagy

Recent literature studies have also suggested that selective clearance of the damaged mitochondria can promote cellular survival, while non-selective mitophagy leads to neural injury. Physiologically, Lazarou et al. (2015) have described that ubiquitin chains would tag protein aggregates and impair the mitochondria to activate selective autophagy in HeLa cells. Then, PINK1 phosphorylates these ubiquitin chains to further modulate the autophagy signals in the mitochondria. The expression of PINK1 maintains a low level in healthy and polarized mitochondria, which is determined by voltage-dependent proteolysis. If the mitochondria sustained damage, PINK1 would rapidly accumulate to OMM and signal the depolarized mitochondria to Parkin, which amplifies the mitophagy cascade (Narendra et al., 2010; Lazarou et al., 2015). PINK1/Parkin-induced mitochondrial clearance provides protection against neuronal injury, especially the CA1 neurons in the hippocampus of cerebral ischemia in rats (Wang H. et al., 2019). The mislocalization of Parkin induced by oxygen hemoglobin in primary neurons resulted in impaired clearance of the damaged mitochondria and reduced neuronal viability (Deng et al., 2021). Interestingly, the highly selective PINK1/Parkin-mediated mitophagy has been confirmed to be monitored by mitochondrial fission (Burman et al., 2017). Fission-associated protein Drp1 KO HEK293 and HeLa cells developed a higher rate of mitophagy and increased the translocation of Parkin to misfolded protein aggregates of the mitochondria-localized mutant ornithine transcarbamylase (ΔOTC). However, with impaired fission, the selectivity of mitophagy to ΔOTC was destroyed, causing enhanced elimination of healthy mitochondria (Burman et al., 2017). In the early stage of ischemic stroke, Drp1-dependent fission was activated, which selectively triggered the autophagy of dysfunctional mitochondria (Zuo et al., 2014). The inhibition of Drp1 counteracted selective mitophagy and caused the accumulation of the damaged mitochondria (Zuo et al., 2014). It seems possible that mitophagy becomes detrimental partially due to its non-selective clearance of healthy mitochondria. Selective mitophagy targets the damaged mitochondria and reduces the release of DAMPs.

In addition to the abovementioned phenomenon and hypotheses, the controversial role of mitophagy may also be due to different upstream stimulatory factors, mitophagy markers, and intervention phases. To eliminate the damaged mitochondria while maintaining healthy mitochondria, future studies will be needed to uncover the mechanism of mitophagy regulation. A clear definition of the marginal value that identifies the appropriate mitophagy may promote the clinical translation of MQC.

In general, the intricate role of mitophagy seems to be associated with the different NVU cells, time courses of stroke, disease severity, mitophagy selectivity, etc. In addition, different protocols of mitophagy intervention, including drug species, routes, time points, and doses of administration, may be responsible for diverse results of studies. Mitophagy-induced downstream cascades could be various in neurons, glial cells, and endothelial cells. Activated mitophagy reduces cell apoptosis and senescence in neurons and endothelia while alleviating inflammatory responses in microglia and astrocytes. Different activities of mitophagy were reported during the development of stroke. Mitophagy can be activated by great mitochondrial damage at the acute stage of stroke, which might be neuroprotective by eliminating the damaged mitochondria. Due to the repair of damage in the subacute and chronic stages, mitophagy could be attenuated. However, extended or immoderate mitophagy in the chronic stage could be harmful due to massive mitochondrial degradation. The severity of stroke also determines the role of mitophagy. Under moderate stress with bits of mitochondrial damage, selective autophagy of mitochondria is protective to eliminate dysfunctional ones and maintain the homeostasis of the mitochondrial network. However, when suffering from prolonged ischemic insult or extensive hemorrhage, dysfunctional mitochondria explode and trigger excessive and unselective mitophagy, leading to severe neural injury.

## The Axes of Mitophagy

**FIGURE 4:**
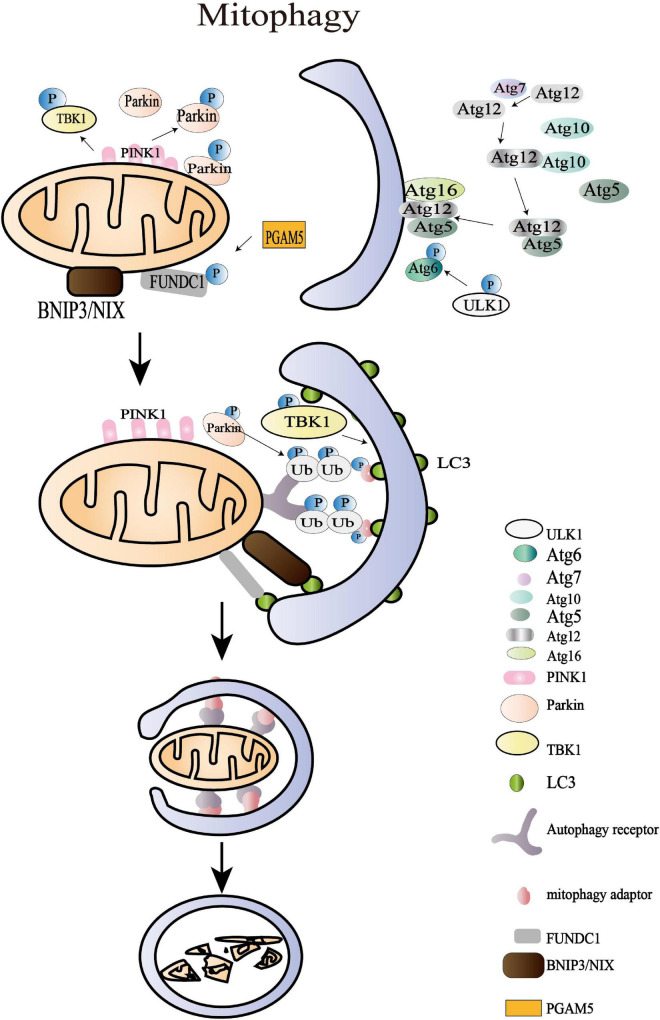
Mitochondrial mitophagy processes. The damaged mitochondria with membrane depolarization initiate OMM protein ubiquitylation at the first stage of mitophagy. Then, the IMM protein PTEN-induced putative kinase protein 1 (PINK1) is accumulated and translocated to OMM, and the damaged mitochondria with membrane depolarization initiate OMM protein ubiquitylation at the first stage of mitophagy. Then, the IMM protein PINK1 is accumulated as a functional full-length form and is translocated to OMM where it triggers the mitophagy pathways through recruiting the E3 ligase Parkin. The amplified ubiquitin signals on the impaired mitochondria subsequently recruit mitophagy adaptors (OPTIN, NBR1, BNIP3, NIX, NDP52, and p62/SQSTM1), which bind to autophagy core units and facilitate the synthesis of autophagosomes. The following processes of engulfing mitochondria are regulated by more than 30 kinds of Atg. The last stage of mitophagy is the autophagosome-lysosome fusion that is modulated by the Atg8 family, consisting of LC3 and GABARAP subfamilies. Apart from the archetypical PINK1/Parkin pathway, BNIP3L/NIX, FUNDC1, AMBRA1, and the lipid receptor Cardiolipin is also involved in mitophagy where it triggers the mitophagy pathways through recruiting the E3 ligase Parkin. The amplified ubiquitin signals on the impaired mitochondria subsequently recruit mitophagy adaptors (OPTIN, NBR1, BNIP3, NIX, NDP52, and p62/SQSTM1), which bind to autophagy core units to facilitate the synthesis of autophagosomes. The following processes of engulfing mitochondria are regulated by more than 30 kinds of autophagy-related proteins (Atg). The last stage of mitophagy is the autophagosome-lysosome fusion that is modulated by the Atg8 family, consisting of LC3 and GABARAP subfamilies. Apart from the archetypical PINK1/Parkin pathway, BNIP3L/NIX, FUNDC1, AMBRA1, and the lipid receptor cardiolipin are also involved in mitophagy.

Mechanically, the damaged mitochondria with membrane depolarization initiate OMM protein ubiquitylation through a PINK1/Parkin pathway at the first stage of mitophagy (Figure 4). The IMM protein PINK1 is accumulated and translocated to OMM, where it triggers the mitophagy pathways through recruiting E3 ligase Parkin (also named as PARK2 or PRKN) (Jin et al., 2010). Yet, in the physiological status, PINK1 is cleaved at Ala103 by PARL protease, which acts as a monitor of mitochondrial health (Lysyk et al., 2020). In addition, a recent study has found that a highly conservative membrane scaffold protein prohibitin 2 (PHB2) mediates mitophagy *via* the PHB2-PARL-PGAM5-PINK1 pathway (Yan et al., 2020). The overexpression of PHB2 stabilizes PINK1, thus promoting mitophagy (Yan et al., 2020). The accumulation of PINK1 leads to the phosphorylation of several OMM proteins, including Mfn2 (Chen and Dorn, 2013), ubiquitin (Lazarou et al., 2015), Miro2 (Wang et al., 2011), and Parkin (Vives-Bauza et al., 2010). This series of PINK1-dependent phosphorylation recruits Parkin to the membrane surface of defective mitochondria and stimulates its ligase activity (Koyano et al., 2014). The overexpression of PINK1 and Parkin was suggested to promote mitochondria collapse and trafficking to the perinuclear area, an autophagy-associated subcellular region (Vives-Bauza et al., 2010). The activated Parkin then catalyzes the ubiquitylation of OMM proteins such as Mfn1, Mfn2, Fis1, voltage-dependent anion-selective channel (VDAC), and CDGSH iron-sulfur domain-containing protein 1 (CISD1) (Deol et al., 2020). These substrates of Parkin lack specificity, but the ubiquitin chains assembling on the substrates are varied in density, structure, linkage, and ubiquitin-binding domains (UBDs) (Harper et al., 2018; Deol et al., 2020). Diverse ubiquitin chains are able to bind specific mitophagy receptors and consequently accurately regulate autophagosomal capture (Harper et al., 2018). However, there are wide gaps in our knowledge of how these ubiquitin chains distribute across different proteins and how they build up complex chain topologies.

The amplified ubiquitin signals on the impaired mitochondria were confirmed to recruit the mitophagy adaptors that bind to autophagy core units and facilitate the synthesis of autophagosomes (Yamano et al., 2020). These mitophagy adaptors, including OPTIN, NBR1, BNIP3, NIX, NDP52, and p62/SQSTM1, have been reported in multiple studies (Kerr et al., 2017; Panigrahi et al., 2020). In HeLa cells, TBK1 is an essential protein in a self-reinforcing positive circle coordinating mitophagy adaptor phosphorylation, mitochondria ubiquitylation, and efficient mitophagy (Heo et al., 2015). The spherical bilayer autophagosome is formed and elongated *de novo* on the target mitochondria, contributing to sequestering oxidized or decayed constituents (Lee et al., 2012). The following processes of engulfing mitochondria are regulated by more than 30 Atg, including Atg1/ULK1 protein-kinase complex, Atg12-Atg8/LC3 conjugated systems, and Atg9Atg2-Atg18 complex, and PI-binding proteins (Tanida, 2011; Lee et al., 2012). The last stage of mitophagy is the fusion of autophagosome and lysosome that is modulated by the Atg8 family consisting of LC3 and GABARAP subfamilies (Nguyen et al., 2016). The mitochondrial contents are then degraded by the lysosomal hydrolases. Apart from the archetypical PINK1/Parkin pathway, BNIP3L/NIX, FUNDC1, AMBRA1, and the lipid receptor Cardiolipin is also involved in mitophagy (Di Rita et al., 2018; Gatica et al., 2018). Beyond the acknowledged role of BNIP3L/NIX in reticulocyte maturation, the overexpression of BNIP3L was shown to rescue mitophagy in Parkin^–/–^ mice after ischemic stroke, which highlighted its independent effect in promoting mitophagy (Yuan et al., 2017). The dimerization of BNIP3L achieves higher efficiency in recruiting autophagosomes and promoting mitophagy (Marinković et al., 2021). Nevertheless, the specific role of these adaptors and receptors in controlling mitophagy during physiological and pathological processes remains to be explored, which may provide new insight into MQC.

## Interaction Between Mitochondrial Dynamics and Mitophagy

Given that the size of mitochondria is bigger than that of autophagosomes, it is reasonable to assume that the sequestration of the damaged mitochondria is a crucial event to facilitate mitophagy (Seabright et al., 2020). The mitochondrial fission protein, Drp1, was shown to regulate the recruitment of Parkin and autophagosome formation (Duan et al., 2020). Fission-induced mitophagy has been reported to be neuroprotective or harmful in stroke. Hypoxia/reoxygenation of primary astrocytes from rats can lead to excessive Drp1 dephosphorylation at Ser 637, thereby increasing mitochondrial fission and redistributing the mitochondria to a smaller size in the perinuclear region (Quintana et al., 2019). This shift in mitochondrial dynamics may be beneficial to maintain neural viability by facilitating mitophagy during metabolic distress (Quintana et al., 2019). In agreement, Zuo et al. (2016) reported that Drp1 not only mediated mitochondrial fission but also recruited the proautophagic proteins to the OMM, which enhanced mitophagy and reduced the accumulation of the damaged mitochondrial fragments in the ischemic region of severe global ischemia rats and hippocampal neurons induced by OGD *in vivo*. Silencing of Drp1 did not change the level of LC3B and p62, but decreased the LC3 positive puncta surrounding the mitochondria (Zuo et al., 2016). However, some studies suggest that after stroke fission/mitophagy activation might be harmful. In the ipsilateral brain tissue of tMCAO-operated rats and the SH-SY5Y cell line stimulated by ONOO^–^, the activation of the Drp1/PINK1/Parkin signaling pathway mediates mitophagy and contributes to brain damage (Feng et al., 2018; Lan et al., 2018). Possible causes of these different effects might be the excessive mitochondrial fission and mitophagy induced by the distinct extension of reperfusion time (Feng et al., 2018).

Fusion is a process that increases the quantity of mitochondrial cristae and the activity of ATP synthase, therefore spares the mitochondria from autophagy (Gomes et al., 2011). Research using MEFs and HeLa cells reported that the fusion between depolarized and polarized mitochondria can dilute and compensate for the damaged mitochondria and obviate mitophagy (Tanaka et al., 2010). In reverse, an increase in the mitophagy protein Parkin can eliminate Mfn1/2 and prevent the fusion of the mitochondria (Tanaka et al., 2010). The dynamic balance between mitophagy and fusion maintains the normal mitochondrial quality and quantity. During the acute phase of ischemic stroke, increased mitophagy was shown to be accompanied by the enhancive fusion markers Mfn1 and Mfn2 on astrocytes (Cao et al., 2021). This cross-talk of fusion and mitophagy inhibited mitochondria-derived oxidative stress, contributing to limited A1 astrocyte polarization and reduced inflammatory cytokines (Cao et al., 2021). Twig et al. (2008) have proven that mitochondrial fission and the following selective fusion filter out dysfunctional mitochondria and promote their elimination by mitophagy.

## Conclusion and Perspective

Mitochondrial quality control plays a fundamental role in the pathophysiology of stroke. The disturbance of mitochondrial homeostasis is an upstream event in NVU dysfunctions after stroke. The altered activity of mitochondrial fission and fusion is associated with mtDNA integrity, oxidative stress, calcium homeostasis, neuroinflammation, and mitochondrial apoptosis. The changes and effects of mitophagy still remain controversial. Proper mitophagy seems to be neuroprotective for its effect on clearing the damaged mitochondria, while excessive mitophagy damages energy generation and mitochondria-associated signal pathways. The balance between mitochondrial dynamics and mitophagy is more crucial than the absolute level of each process.

Though multiple advances have been made in the mechanism of mitochondrial dynamics and mitophagy after stroke, this is still a complexity that requires more research. Current studies might be limited by the lack of reliable methods to assess the status of MQC and pharmacologic modulators to specifically regulate it. The detailed mechanism of the interaction between mitochondrial dynamics and mitophagy remains to be explored, which can provide valuable insights into stroke treatment. The development of circulating plasma or cerebrospinal fluid biomarkers linked to stroke injury is beneficial for identifying the intricate role of MQC. Genome, transcriptome, proteome, and epigenome sequencing techniques can identify the molecular heterogeneity that reveals the feature of MQC in a patient-specific manner, which may exploit novel frontiers for therapeutic interventions in stroke.

## Author Contributions

MY was the first author of this review who referred the relevant literature and completed the first draft of this article. YH was the co-author participated in the analysis of the document and modification of this article. SD, LX, MT, YX, and CL modified the draft and figures. FZ and YG are the designers who are in-charge of the project and direct this review’s writing. All authors agreed with the final text.

## Conflict of Interest

The authors declare that the research was conducted in the absence of any commercial or financial relationships that could be construed as a potential conflict of interest.

## Publisher’s Note

All claims expressed in this article are solely those of the authors and do not necessarily represent those of their affiliated organizations, or those of the publisher, the editors and the reviewers. Any product that may be evaluated in this article, or claim that may be made by its manufacturer, is not guaranteed or endorsed by the publisher.
